# Excess Mortality due to natural causes among whites and blacks during the COVID-19 pandemic in Brazil

**DOI:** 10.1590/0037-8682-0283-2021

**Published:** 2022-01-28

**Authors:** Renato Azeredo Teixeira, Ana Maria Nogales Vasconcelos, Ana Torens, Elisabeth Barboza França, Lenice Ishitani, Ana Luiza Bierrenbach, Daisy Maria Xavier de Abreu, Fátima Marinho

**Affiliations:** 1 Universidade Federal de Minas Gerais, Faculdade de Medicina, Programa de Pós-Graduação em Saúde Pública, Belo Horizonte, MG, Brasil.; 2 Vital Strategies, New York, United States of America.; 3 Universidade de Brasília, Departamento de Estatística, Brasília, DF, Brasil.; 4 Universidade Federal de Minas Gerais, Grupo de Pesquisas em Epidemiologia e Avaliação em Saúde, Belo Horizonte, MG, Brasil.; 5 Hospital Sírio-Libanês, Instituto de Ensino e Pesquisa, São Paulo, SP, Brasil.

**Keywords:** Excess mortality, COVID-19, Race, Skin color, Health information system

## Abstract

**INTRODUCTION:**

Excess Mortality by all causes considers deaths directly related to COVID-19 and those attributed to conditions caused by the pandemic. When stratified by social dimensions, such as race/color, it allows for the evaluation of more vulnerable populations. The study estimated the excess mortality by natural causes, separating the white and black populations in 2020.

**METHODS:**

Public civil registration data on deaths observed in 2020, corrected for under registration, were used. The expected number of deaths was estimated based on the mortality rates observed in 2019, applied to the estimated population in 2020. The difference between the values expected and observed and the proportion of excess was considered the excess mortality.

**RESULTS:**

The present study found an excess of 270,321 deaths (22.2% above the expected) in 2020. Every state of Brazil reported deaths above the corresponding expected figure. The excess was higher for men (25.2%) than for women (19.0%). Blacks showed an excess of 27.8%, as compared to whites at 17.6%. In both sexes and all age groups, excess was higher in the black population, especially in the South, Southeast, and Midwest regions. São Paulo, the largest in population number, had twice as much excess death in the black population (25.1%) than in the white population (11.5%).

**CONCLUSIONS:**

The present study showed racial disparities in excess mortality during the COVID-19 pandemic in Brazil. The higher excess found for the black suggests an intrinsic relationship with the socioeconomic situation, further exposing the Brazilian reality, in which social and structural inequality is evident.

## INTRODUCTION

The coronavirus disease 2019 (COVID-19) pandemic, declared an internationally important public health emergency in January 2020 by the World Health Organization (WHO)^1^ and a national important public health emergency by the Brazilian Ministry of Health^2^ in February 2020, caused more than 7.6 million cases and approximately 195,000 notified deaths in 2020^3^ in Brazil. These numbers are expressive and place Brazil as the second country in terms of number of cases and deaths, behind only the United States^4^. 

Confirmation of COVID-19 as the cause of death in Brazil depends mostly on positive result in the exam for the detection of the genetic material of SARS-CoV-2 (RT-PCR - polymerase chain reaction). In the early months of the pandemic, given the low testing capacity almost everywhere, the Brazilian government prioritized the testing of hospitalized patients with a suspicion of COVID-19 and, in some states, instituted sample collection after death for this specific exam^5^. In addition to the low testing capacity, other problems, such as difficulty in collecting, storing, and transporting samples in a timely manner, indicate the existence of under-reporting of cases and deaths in the country. Therefore, the real number of underlying causes of the deaths can hardly be obtained by using crude mortality data from local health information systems^6^.^.^

Estimating COVID-19 specific mortality is not a Brazilian exclusive problem^7^. Difficulties in accurately establishing the underlying cause of death make mortality by all causes an important indicator of the magnitude of the impact of the epidemic in the population mortality. This occurs because, apart from the disease causing an elevated number of direct deaths *per se*, it also affects the number of indirect deaths due to other causes, as a consequence of the overburdening of the health system, population fear of seeking out medical care for sudden illnesses, as well as chronic conditions due to the risk of SARS-CoV-2 infection being high in hospitals and ambulatory facilities, restrictions on people’s circulations, among other problems^8^. Hence, since all-cause mortality is a simple indicator that depends solely on the count of the total number of deaths, which is a type of data potentially faster to obtain, it becomes an essential indicator to aid in decision-making and the design of actions to control epidemics. This indicator can be extremely useful in a context of social and economic inequalities, where the poorest population and those living in difficult to reach regions have very low access to quality health care, with a subsequent impact on the profile of their causes of death^9^. In a crisis scenario such is the current one with the COVID-19 pandemic, the uneven access to diagnoses and to quality care for the sick and dying are likely reflected in an uneven mortality risk. In this sense, information stratified by social dimensions, such as race/color, becomes important in the evaluation of the differential impact of COVID-19 on more vulnerable populations^10^. The objective of the present study is to estimate excess mortality by natural causes in the white and black populations, according to age and sex, in Brazil and its states in 2020. 

## METHODS

### Data sources

This observational study used, as one of its data sources, the Mortality Information System (SIM, in Portuguese) of the Brazilian Ministry of Health, which is responsible for collecting, storing, and distributing information on deaths in the Brazilian territory. The SIM is the main source of data about deaths in the country and follows a standardized process from data collection to several data quality checks leading to the selection of the underlying cause of death^11^ ^-^ ^12^. However, the SIM has a delay of approximately two years until it finally publishes the official data. Given the unavailability of the official SIM data for 2020, our study had to use the data from the Civil Registry (CR) for this year, made available by the Association of Natural Persons Registers (ARPEN-Brazil), through the Transparency Website^13^. The CR data, from ARPEN website, provides data on mortality by natural causes in Brazil since 2019. 

Although the SIM and the CR databases are distinct, the Death Certificate (DC) is the common collection instrument of both systems. In Brazil, the DC is usually filled out by the attending physician and, less commonly, by coroners and pathologist. After completion, three copies of the DC are sent out: the first goes to the data processing section of the SIM, usually the municipal Health Secretariat; the second to the family of the deceased so that it can be officially recorded and filed at the notary public office, which is a requirement for burial and other bureaucratic needs; and the third copy should remain at the notifying institution (hospital, other health establishment, coroner's office, or SVO) to be included in the medical records of the deceased. This procedure may vary according to whether the death was due to natural or external causes^14^. 

In this study we used both the SIM and the ARPEN data. The SIM had to be used so as to correct for under-reporting as explained below. It is important to emphasize that the data from ARPEN used in this work refer to deaths by natural causes, identified as such in the section "type of death” (natural or external cause). For compatibility purposes, DCs issued by the coroner's office were excluded from the SIM, so that the definitions of death from natural causes were as compatible as possible in the two databases. 

### Correction of under-reporting in the CR

Given that the registered deaths are inferior in number in the CR data as compared to the SIM in most states of Brazil^15^, we used a correction factor to account for the numeric differences of the two sources in 2020, according to the numbers observed in both sources in 2019, separately for each federal unit, sex, and age. For example, if 100 deaths and 120 deaths were respectively registered in CR and SIM in 2019 for males over 80 years of age in the state of Rio de Janeiro, the correction factor used in 2020 for this stratum was 1.2. Only after this correction was it possible to compare the CR data for 2020 with the expected deaths data for this year. For the state of São Paulo, the opposite situation was observed in 2019, that is, the SIM had higher numbers than the CR. For this reason, the correction made for this state had to be different from the others, having applied a correction factor of less than 1, so that the data from both databases were comparable.

As regards the variable race/color, over 17% of records of the CR data have missing values. Meanwhile, in the SIM data, less than 3% have missing values on recent years. To avoid discarding CR observations with missing values on race/color, a proportional redistribution was performed, based on the proportion of deaths by race/color from the SIM, according to sex, age, and federal unit. 

### Level of Analysis

In order to show the local realities, this study considered information according to sex, age and race/color in the 27 federal units (26 states plus the federal district), for each epidemiological week (EW), according to the date of death. The following age groups were used: 0 to 29, 30 to 59, 60 to 79, and 80 years of age and older. As race/color categories, “white” and “black” (represented by the sum of blacks and browns, called ‘pardos’ in Portuguese) were used, which represent 99% of all deaths with information on race/color. 

### Excess mortality

The indicator “excess mortality” took into consideration the difference between the observed values and the expected values at the most disaggregated level, in other words, according to sex, age group, race/color, EW, and state. The more aggregated final estimates were obtained from the sum of those more disaggregated levels. The “expected deaths” were estimated using the mortality rates observed in 2019 and applied to the 2020 population. For example, in 2019, 77 deaths were observed in epidemiological week 20 among 30- to 59-year-old white females in Minas Gerais, which results in a rate of 4.2 deaths per 100,000 inhabitants (population of 1,811,637). Applying this rate in the population of 2020, we expected 78 deaths for this year (population of 1,827,084 inhabitants in 2020). The population data estimated by race/color was obtained using the proportions observed in the microdata of the National Household Sample Survey (PNAD)^16^. The PNAD is a periodic structural survey conducted by the Brazilian Institute of Geography and Statistics (IBGE), which aims to monitor the evolution of the workforce and other information necessary for the study of the country's socioeconomic development, by social analysts and public policymakers. The microdata was applied to the population projections by the IBGE - the official intercensal population estimates^17^. The “observed deaths” refer to the CR data for 2020, to which we applied the correction factors mentioned above. The positive difference between the observed values and the expected values by EW, for 2020, were considered as “excess deaths”. The excess mortality proportion was calculated as the ratio of the excess and the expected values, multiplied by 100. A sensitive analysis using Poisson regression is presented in the  Supplementary Material Tables 1-4.

## RESULTS

**FIGURE 1:**
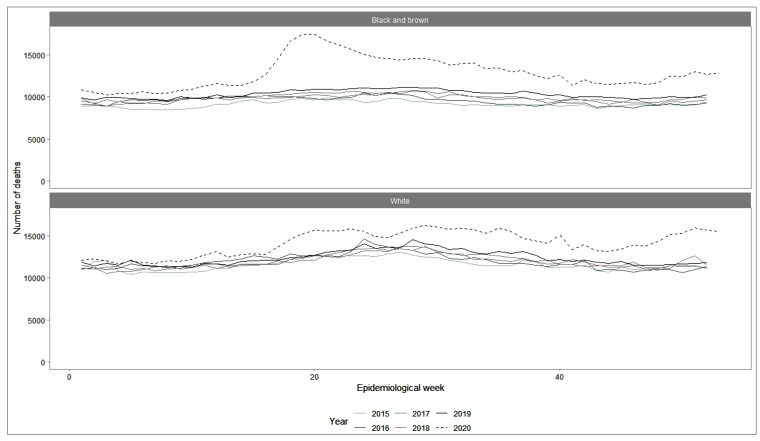
Number of observed deaths per week according to race/color. Brazil, 2015 to 2020.**Data sources:** SIM between 2015 and 2019 and CR (ARPEN) in 2020.

In Brazil, from EW 12 (March 15^th^, 2020) on, there is a sustained excess of deaths, intensified dramatically from EW 17 (April 19th) on. This excess reached its peak between May 3rd and 16^th^, with over 32,000 deaths for both whites and for blacks, including over 17,000 blacks and over 15,000 whites (Figure 1).

Table 1 shows the number of excess deaths in the country, its regions, and its states, according to race/color. For 2020, 1,216,296 deaths by natural causes were expected in the country. This number represents a 22% increase from the amount expected for the year, which means an excess of 270,321 deaths. When analyzed by race/color, blacks showed higher values in comparison to the white population, with 27.8% (n=153,284) deaths above the expected number, while whites reached 17.6% (n=117,037) above the expected figure. 

**TABLE 1:** Number of deaths expected and in excess, and proportion of excess mortality, according to race/color. Brazil, regions, and states, 2020.

Location	White			Black			White+Black		
	Expected	Excess	%	Expected	Excess	%	Expected	Excess	%
**Midwest**	**35,794**	**10,847**	**30.3**	**42,139**	**14,125**	**33.5**	**77,932**	**24,972**	**32.0**
Federal District	6,725	2,136	31.8	6,858	2,836	41.4	13,583	4,972	36.6
Goiás	15,798	4,418	28.0	18,510	5,501	29.7	34,307	9,918	28.9
Mato Grosso do Sul	7,251	1,974	27.2	7,269	2,206	30.3	14,520	4,180	28.8
Mato Grosso	6,020	2,319	38.5	9,502	3,583	37.7	15,522	5,901	38.0
North	15,834	7,515	47.5	57,036	21,558	37.8	72,870	29,073	39.9
Acre	720	535	74.3	2,860	828	29.0	3,580	1,363	38.1
Amazonas	2,770	1,569	56.7	12,320	5,782	46.9	15,090	7,351	48.7
Amapá	712	407	57.2	2,247	1,092	48.6	2,959	1,500	50.7
Pará	6,624	2,956	44.6	28,792	9,792	34.0	35,416	12,748	36.0
Roraima	2,752	973	35.4	4,435	1,601	36.1	7,187	2,574	35.8
Rondônia	425	283	66.7	1,515	933	61.6	1,940	1,217	62.7
Tocantins	1,832	791	43.2	4,867	1,529	31.4	6,700	2,320	34.6
Northeast	87,272	23,157	26.5	225,487	59,306	26.3	312,760	82,463	26.4
Alagoas	4,863	1,375	28.3	12,923	3,699	28.6	17,786	5,074	28.5
Bahia	17,989	3,517	19.5	61,695	12,957	21.0	79,684	16,474	20.7
Ceará	13,863	4,843	34.9	36,392	13,310	36.6	50,255	18,152	36.1
Maranhão	6,567	2,914	44.4	25,801	9,196	35.6	32,367	12,111	37.4
Paraíba	7,739	2,017	26.1	16,780	2,822	16.8	24,519	4,839	19.7
Pernambuco	20,021	4,773	23.8	36,782	10,340	28.1	56,803	15,114	26.6
Piauí	4,325	1,045	24.2	15,495	2,474	16.0	19,820	3,519	17.8
Rio Grande do Norte	8,434	1,744	20.7	10,979	2,363	21.5	19,414	4,107	21.2
Sergipe	3,471	930	26.8	8,640	2,143	24.8	12,111	3,073	25.4
South	165,559	19,681	11.9	25,963	5,914	22.8	191,522	25,595	13.4
Paraná	54,907	7,350	13.4	13,181	3,172	24.1	68,088	10,522	15.5
Rio Grande do Sul	74,490	6,870	9.2	9,507	1,734	18.2	83,997	8,605	10.2
Santa Catarina	36,162	5,461	15.1	3,275	1,008	30.8	39,438	6,469	16.4
Southeast	360,159	55,837	15.5	201,052	52,381	26.1	561,212	108,218	19.3
Espírito Santo	10,795	2,672	24.8	10,951	4,001	36.5	21,746	6,672	30.7
Minas Gerais	67,317	11,269	16.7	62,374	11,347	18.2	129,691	22,616	17.4
Rio de Janeiro	72,434	17,698	24.4	58,203	19,596	33.7	130,638	37,294	28.5
São Paulo	209,613	24,198	11.5	69,524	17,438	25.1	279,136	41,636	14.9
Brazil	664,618	117,037	17.6	551,678	153,284	27.8	1,216,296	270,321	22.2

Every state reported an excess of deaths above the expected figure for 2020, ranging from 10.2% in Rio Grande do Sul to 62.7% in Rondônia. The Southeast, Northeast, and North regions were the most affected. In 2020, there were 108,218 (19.3% excess) deaths above the expected figure; in the Northeast, the number was 82,463 deaths, 26.4% excess deaths; and in the North region, there were 29,043 deaths, corresponding to 39.9% excess deaths, the highest among the Brazilian regions. 

There was a higher excess mortality for blacks with higher differences in proportions among the population groups of the Southeast (26.1% among blacks and 15.5% among whites), and South (22.8% among blacks and 11.9% among whites). In the North region, excess mortality was higher among the white population: 37.8% (n=21,558) and 47.5% (n=7,515) of excess deaths among the black and white populations, respectively. In the Northeast region, the proportion of excess was similar for the two groups, reaching 26.5% for whites and 26.4% for blacks.

Among the states, São Paulo stands out for showing not only the highest absolute numbers of excess deaths, but also for having twice as many excess deaths among the blacks (25.1%, n= 17,438) than among whites (11.5%, n= 24,198).

From 2015 to 2019, approximately 52% of the deaths by natural causes in the country happened among men (data not presented). The excess deaths observed in 2020 were also higher among men. Men reached a total of 25.2% (n=159,157) of excess deaths, while women reached 19.0% (n=111,164). For both sexes, the excess deaths were higher among the black population. The numbers of excess deaths were approximately 31.0% (n=93,200) for black men and 23.9% (n=60,084) for black women, while for white men and women, the excess was 20.0% (n=65,957) and 15.3% (n=51,080), respectively (Table 2).

**TABLE 2:** Proportional excess mortality according to race/color and sex. Brazil, regions, and states, 2020.

Location	White		Black	
	Women	Men	Women	Men
**Midwest**	**26.0**	**34.4**	**30.3**	**36.0**
DF	23.9	40.3	35.5	46.1
GO	24.5	31.3	27.3	31.5
MS	23.1	31.1	27.6	32.4
MT	37.1	39.6	34.3	40.1
North	44.3	50.4	33.5	40.9
AC	76.7	72.3	31.8	27.0
AM	50.5	62.9	42.1	50.3
AP	50.1	65.0	44.8	51.5
PA	41.3	47.9	28.3	38.2
RO	34.1	36.4	34.6	37.1
RR	53.7	79.2	63.1	60.6
TO	43.7	42.7	29.0	33.2
Northeast	23.4	30.2	21.8	30.2
AL	23.3	34.3	22.4	34.4
BA	17.2	22.2	18.5	23.2
CE	32.5	37.8	28.5	43.8
MA	39.5	49.1	29.9	39.9
PB	22.3	30.7	13.9	19.4
PE	19.9	28.7	22.7	33.0
PI	21.9	26.7	14.6	17.1
RN	19.0	22.5	19.3	23.4
SE	26.2	27.4	20.8	28.2
South	9.7	14.0	21.9	23.5
PR	10.9	15.6	21.4	26.1
RS	7.8	10.6	19.0	17.6
SC	11.9	18.0	32.6	29.2
Southeast	13.4	17.7	22.8	28.9
ES	23.8	25.7	28.0	43.6
MG	15.9	17.6	17.1	19.1
RJ	18.4	31.3	26.5	40.8
SP	10.2	12.9	23.8	26.1
Brazil	15.3	20.0	23.9	31.0

The highest portion of deaths occurring in 2020 was concentrated in the age groups of 60 -79 years and 80 years and above. Excess deaths were higher for blacks than for whites in all age groups. In the younger age groups, Brazil had an excess of deaths among blacks of 32.9% (n=11,994) among those individuals younger than 29 years of age, and of 37.0% (n=46,996) in the age group of 30 to 59 years, as compared to 22.6% (n=5,757) and 31.1% (n=29,701) of excess deaths in the white population, for the same age groups, respectively (Table 3). 

**FIGURE 2:**
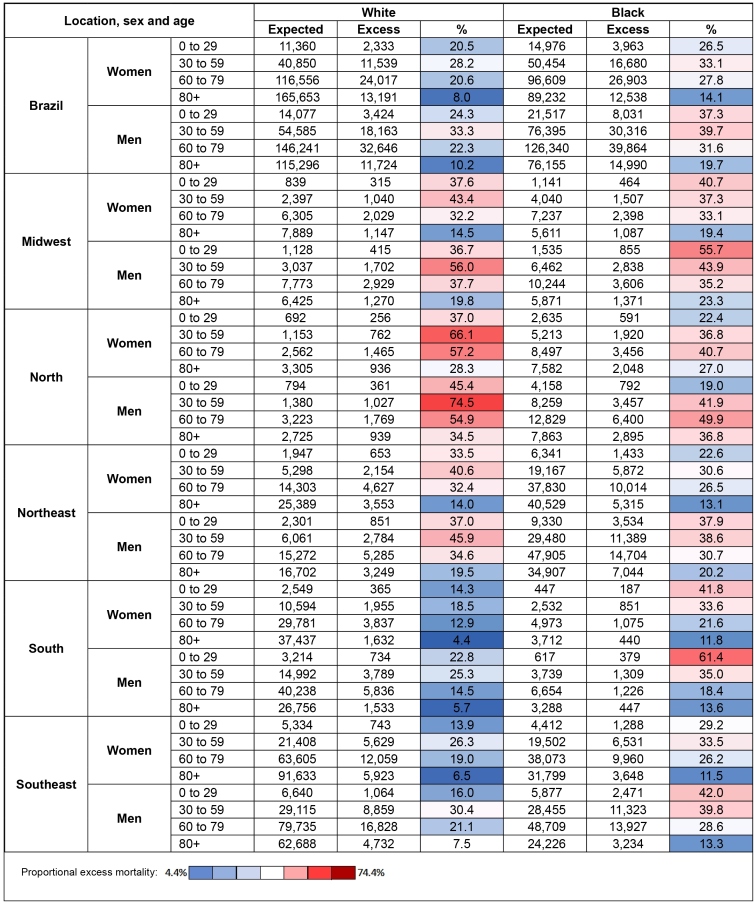
Number of deaths expected and in excess, together with proportional excess mortality, according to race, sex, and age groups. Brazil and regions, 2020.

When comparing the excess mortality between categories of race/color according to sex and age in the country, the highest difference was among elderly men, with almost twice as much excess deaths among blacks, at 19.7% (n=14,990), as compared to 10.2% (n=11,724) among whites (Figure 2). According to region, the excess mortality of young males also stands out in the South and Southeast regions, with a 2.6-fold higher proportion for blacks. 

**TABLE 3:** Proportional excess mortality according to race and age groups. Brazil, regions, and states, 2020.** **

Location	White				Black			
	0 to 29	30 to 59	60 to 79	80+	0 to 29	30 to 59	60 to 79	80+
**Midwest**	**37.1**	**50.5**	**35.2**	**16.9**	**49.3**	**41.4**	**34.3**	**21.4**
Federal District	41.0	46.9	38.6	18.7	59.6	51.0	39.3	28.7
Goiás	22.0	49.4	35.3	14.2	32.8	35.7	33.4	18.3
Mato Grosso do Sul	61.5	39.7	28.7	16.3	88.6	45.6	24.5	17.0
Mato Grosso	39.7	68.2	39.1	23.4	46.1	41.7	40.5	26.6
North	41.5	70.6	55.9	31.1	20.4	39.9	46.2	32.0
Acre	79.0	115.5	67.5	61.0	33.3	35.5	32.3	17.3
Amazonas	23.7	75.9	73.0	42.1	14.7	49.4	58.5	45.7
Amapá	54.9	85.7	58.8	41.8	16.7	58.0	58.6	46.3
Pará	34.6	63.0	58.2	27.4	15.9	32.5	44.2	29.3
Roraima	58.2	67.3	33.7	19.3	49.3	50.3	34.5	21.5
Rondônia	69.3	98.8	67.7	49.4	59.0	65.4	61.7	58.1
Tocantins	58.8	63.0	51.1	29.1	31.5	32.3	37.4	24.2
Northeast	35.4	43.5	33.5	16.2	31.7	35.5	28.8	16.4
Alagoas	34.2	40.7	38.0	14.6	43.8	39.5	29.1	15.8
Bahia	20.6	34.3	25.5	12.1	12.8	31.4	24.9	11.6
Ceará	27.0	50.5	45.2	25.9	54.6	43.8	42.0	24.9
Maranhão	32.5	61.4	54.6	31.5	22.7	35.5	43.5	31.2
Paraíba	47.9	47.8	29.3	16.7	32.6	28.6	16.3	8.5
Pernambuco	56.6	41.7	31.1	10.3	65.6	44.1	25.7	13.0
Piauí	45.1	37.2	26.8	16.5	16.1	21.8	19.0	9.4
Rio Grande do Norte	26.8	36.9	25.7	12.1	24.0	31.1	19.3	17.2
Sergipe	29.2	50.8	32.0	15.3	21.0	31.9	29.6	14.6
South	19.1	22.5	13.8	4.9	53.2	34.4	19.8	12.7
Paraná	23.2	24.9	15.6	4.9	58.3	37.7	22.1	11.0
Rio Grande do Sul	14.6	19.1	10.9	3.7	49.2	26.3	14.9	12.3
Santa Catarina	19.2	24.5	17.2	7.8	48.2	43.2	25.8	21.6
Southeast	15.1	28.7	20.2	6.9	36.5	37.2	27.5	12.3
Espírito Santo	36.9	44.8	31.1	12.8	55.0	52.2	36.5	18.8
Minas Gerais	19.2	25.8	20.7	10.3	22.1	26.3	20.8	8.7
Rio de Janeiro	42.7	44.7	31.0	11.7	62.1	49.7	32.5	16.1
São Paulo	5.7	24.0	15.7	3.7	21.9	34.4	27.5	11.9
Brazil	22.6	31.1	21.6	8.9	32.9	37.0	29.9	16.6

## DISCUSSION

The present study reveals important racial disparities in excess mortality during the COVID-19 pandemic in Brazil, with a heterogeneous impact among states, sexes, and ages. The total excess mortality was 270,000 deaths, and the excess was 1.6-fold higher among blacks when compared to whites. Among elderly men, blacks had almost twice as many deaths as whites. The state of São Paulo stood out for its high disparity between blacks and whites, 25.1% and 11.5% (ratio of 2.17), respectively. Other studies have also shown that the impacts of mortality by COVID-19 are unequal among populations, especially in terms of socioeconomic and sociodemographic conditions^18^ ^,^ ^19^. 

Excess deaths allow us to quantify the general impact of the COVID-19 pandemic in national mortality, regardless of cause of death. This indicator considers not only the deaths which reported COVID-19 as the declared cause, but also the deaths by COVID-19 which were not diagnosed and the deaths by other causes that can be attributed to the conditions caused by the crisis generated by the pandemic^20^. The comparative analysis at disaggregated levels, considering regionalized, demographic information, enables the identification of the most affected populations and, together with other relevant sources of data, can be used to aid in directing actions to control and prevent the disease in the country.

The distribution of the Brazilian population by race/color differs from region to region and according to state. In general, more than half of the Brazilian population is black (56% in 2019). This percentage varies from 26% in the South to 79.5% in the North of the country. In terms of the age structure, the white population is older than the black, and far more numerous for individuals of 80 years of age and older^16^ ^,^ ^17^. In this study, however, when the excess mortality according to race/color in Brazil was analyzed, a higher excess among the black was found in all of the studied age groups. The highest differentials of excess death between the categories of race/color were in the extreme age groups, children and young adults (<29 years) and the elderly (80 years and over)^21^.

The higher excess mortality in the white population in the North region of the country was unexpected, but could eventually be attributed to behavioral factors that are characteristic of that population group. It is important to bear in mind that the population on these states is mostly composed of what we are here denominating blacks, but in truth are a mix of Indians and other color/races. In general, the white population is a minority with a much higher income, more conditions to travel (international trips, for instance), and their COVID cases with more access to the small number of hospitals available in the region with higher probability of being notified to the CR registry. 

It is important to mention that the states that have mainly white populations had a smaller proportion of excess deaths (Rio Grande do Sul, São Paulo, Paraná, and Santa Catarina). However, the excess mortality was much greater among the black population in those states and was more than double in São Paulo. If on the one hand we can attribute the lower percentages of excess mortality to better access to prevention and medical care in those states, on the other hand, the inequalities according to race/color are evident, with a far higher risk of death among blacks. The wide and complex racist effect involves three dimensions: personal - feelings and behavior, including inferiority/superiority and acceptance/refusal; interpersonal - actions and omissions manifested in the lack of respect, persecution, devaluing, and negligence when dealing with racism and its impacts; and institutional - reduced access to quality services, less access to information, and scarcity of resources and social control^22^. It is important for the racial differences to be highlighted so that societies can face this problem and can intervene in the factors which compromise the access to and use of health services in more egalitarian terms. 

The higher excess mortality identified for blacks suggests an intrinsic relationship between race/color and lower socioeconomic status, vastly observed in other studies. In New York, the numbers of admissions to hospitals and of in-hospital deaths from COVID-19 were higher in neighborhoods with a higher proportion of racial/ethnic minorities, in families living in poverty, and those individuals with a lower level of education^23^. Higher rates of mortality were also observed in counties with a higher percentage of people who identified themselves as African-American (IRR, 1.02; 95% CI, 1.02-1.03; P <0.001) and other racial/ethnic minorities^24^. In the city of São Paulo, Ribeiro et al.^19^ observed a higher rate of mortality among blacks, which was 1.77-fold higher than in whites, and verified a gradient of this disparity as the socioeconomic indicators became worse, especially for people under 60 years of age. Moreover, in Brazil, Baqui et al.^25^ not only noticed a higher risk of death in hospitals for blacks in comparison to whites, but also the category “colored” was the second most important death risk after increasing age. 

In Brazil, 70% of people below the poverty line (internationally defined by the World Bank as US$ 1.90 per day) are black or biracial, although 56.3% of the total population declare themselves as being of that race/color^26^. This finding makes people more likely to live in overcrowded conditions, in multi-generational families, which makes it harder to maintain social distancing^27^. The higher inadequacy of homes found among blacks, including sanitation problems, also determines a higher difficulty to apply adequate prevention habits, like proper hand hygiene^26^.

Another factor to be considered is that this population often works in informal jobs^26^; they depend on jobs which cannot be done remotely^27^. Even the people who can stay at home most likely live with someone who is an essential worker; therefore, they are more likely to be exposed to infection by SARS-CoV-2^28^ ^,^ ^29^. Moreover, they are more likely to use (and for longer distances) public transportation, which also exposes them to a higher risk of contamination^27^.

The fact that this population has less education might make it more difficult for them to understand messages about prevention, symptoms, and ways of disseminating COVID-19^24^. Adding this to the other problems, the black population shows a higher prevalence of chronic comorbidities, such as hypertension^30^, diabetes^31^, and obesity^32^, all risk factors that can result in worse outcomes for COVID-19^24^. The greater difficulty in access to health services^33^ also contributes to having more serious complications of COVID-19, as they seek out medical care too late.

This study has some limitations, as it used two systems as a data source. It is well-known that the data from SIM undergoes a rigorous quality control process at all levels. This makes data more reliable, but it also makes it harder to develop studies capable of providing fast feedback in the course of a pandemic, since it takes nearly two years for SIM data to become available. Therefore, even considering that two sources of data were used, the analysis of the excess death estimates conducted in the present study were considered opportune and helped call attention to the racial disparities in the country. 

Another limitation that should be highlighted is related to the methodology for the correction of the missing data on color/race, an essential step in creating the correction factors of the underreporting of the CR. Even though methods were applied to treat data at the state level, taking into consideration local characteristics, the quality of the generated information is unequal among the states^15^. For the redistribution of the missing data, we followed the premise that the data missing from the CR was equal to that of the registries filled out by the SIM for Brazil; values were differentiated when different strata were considered, especially concerning small numbers. It should also be emphasized that this redistribution took into consideration the state, sex, and age groups to contemplate the local differences. 

Even though life is a fundamental right guaranteed by law in Brazil, with no distinction of any kind, the universal and integral guarantee of health services as an obligation of the State (with no prejudice regarding origin, race, sex, age, or any other form of prejudice) is a different reality. Disparities in mortality according to race /color are evidence of the structural inequalities which exist in Brazil; access to health is divergent and, consequently, the right to life is unequal. Considering the disparity in mortality related to the COVID-19 pandemic, the present study helped to expose the Brazilian reality even further, a reality in which “the color of the skin is a marker of difference”^34^.
