# Supplementary material for: Excess Mortality due to natural causes among whites and blacks during the COVID-19 pandemic in Brazil
Source: Rev Soc Bras Med Trop. 2022 Jan 28;55(Suppl 1):e0283-2021. doi: 10.1590/0037-8682-0283-2021 (PMC9009425; doi:10.1590/0037-8682-0283-2021)
Supplement: Supplementary file 1 [file 1678-9849-rsbmt-55-s01-e0283-2021-supp1.pdf]

Supplementary Material Table 1 – Proportional deaths in states by colors in SIM. Brazil, 2019.

State	Black and Brown	White	Other
Acre	76.8%	19.3%	4.0%
Alagoas	72.3%	27.2%	0.5%
Amazonas	76.5%	17.2%	6.3%
Amapá	75.0%	23.7%	1.3%
Bahia	77.1%	22.4%	0.5%
Ceará	72.2%	27.5%	0.4%
Distrito Federal	50.4%	49.0%	0.7%
Espírito Santo	50.2%	49.4%	0.4%
Goiás	53.7%	45.7%	0.6%
Maranhão	78.8%	20.0%	1.1%
Minas Gerais	47.9%	51.6%	0.5%
Mato Grosso do Sul	48.3%	48.0%	3.8%
Mato Grosso	59.9%	37.8%	2.4%
Pará	80.7%	18.5%	0.8%
Paraíba	68.1%	31.3%	0.5%
Pernambuco	64.5%	35.0%	0.5%
Piauí	77.9%	21.7%	0.4%
Paraná	19.2%	79.7%	1.1%
Rio de Janeiro	44.6%	55.2%	0.2%
Rio Grande do Norte	56.4%	43.2%	0.4%
Rondônia	61.0%	37.7%	1.3%
Roraima	65.1%	18.3%	16.6%
Rio Grande do Sul	11.3%	88.4%	0.3%
Santa Catarina	8.3%	91.4%	0.3%
Sergipe	71.2%	28.5%	0.3%
São Paulo	24.6%	73.9%	1.4%
Tocantins	71.5%	26.9%	1.6%

Supplementary Material Table 2 – Summary of the total correction factors in states used to correct CR data based on SIM. Brazil, 2019.

State	Black and Brown	White
Acre	1.31	1.08
Alagoas	1.07	1.34
Amazonas	1.13	1.40
Amapá	1.24	1.20
Bahia	1.18	1.19
Ceará	1.28	1.35
Distrito Federal	1.00	1.00
Espírito Santo	1.00	1.00
Goiás	1.14	1.10
Maranhão	1.77	1.73
Minas Gerais	1.08	1.08
Mato Grosso do Sul	1.00	1.00
Mato Grosso	1.13	1.15
Pará	1.59	1.43
Paraíba	1.01	1.00
Pernambuco	1.00	1.00
Piauí	1.52	1.56
Paraná	1.06	1.05
Rio de Janeiro	1.00	1.00
Rio Grande do Norte	1.28	1.19
Rondônia	1.05	1.02
Roraima	1.00	1.01
Rio Grande do Sul	1.01	0.99
Santa Catarina	1.08	0.99
Sergipe	1.12	1.07
São Paulo	0.91	0.93
Tocantins	1.39	1.40

Supplementary Material Table 3 – Sensitivity analysis: Expected deaths, excess deaths, and proportional excess deaths in white, black, and brown populations by methods*. Brazil and states, 2020.

States	Black and Brown					
	2019 rate applied to 2020 population			Poisson model		
	Expected deaths	Excess deaths	% excess	Expected deaths	Excess deaths	% excess
Acre	2,860	828	29.0%	3,093	725	23.4%
Alagoas	12,923	3,699	28.6%	12,681	3,776	29.8%
Amazonas	12,320	5,782	46.9%	12,963	5,300	40.9%
Amapá	2,247	1,092	48.6%	2,510	984	39.2%
Bahia	61,695	12,957	21.0%	62,339	12,156	19.5%
Ceará	36,392	13,310	36.6%	37,320	12,541	33.6%
Distrito Federal	6,858	2,836	41.4%	7,224	2,552	35.3%
Espírito Santo	10,951	4,001	36.5%	11,091	3,864	34.8%
Goiás	18,510	5,501	29.7%	18,889	5,206	27.6%
Maranhão	25,801	9,196	35.6%	26,762	8,446	31.6%
Minas Gerais	62,374	11,347	18.2%	65,013	9,025	13.9%
Mato Grosso do Sul	7,269	2,206	30.3%	7,308	2,149	29.4%
Mato Grosso	9,502	3,583	37.7%	9,935	3,251	32.7%
Pará	28,792	9,792	34.0%	29,333	9,409	32.1%
Paraíba	16,780	2,822	16.8%	16,714	2,705	16.2%
Pernambuco	36,782	10,340	28.1%	36,439	10,259	28.2%
Piauí	15,495	2,474	16.0%	15,915	2,191	13.8%
Paraná	13,181	3,172	24.1%	13,652	2,880	21.1%
Rio de Janeiro	58,203	19,596	33.7%	61,199	16,907	27.6%
Rio Grande do Norte	10,979	2,363	21.5%	11,129	2,201	19.8%
Rondônia	4,435	1,601	36.1%	4,716	1,413	30.0%
Roraima	1,515	933	61.6%	1,907	773	40.5%
Rio Grande do Sul	9,507	1,734	18.2%	10,340	1,340	13.0%
Santa Catarina	3,275	1,008	30.8%	3,642	840	23.1%
Sergipe	8,640	2,143	24.8%	8,736	2,075	23.8%
São Paulo	69,524	17,438	25.1%	71,639	15,556	21.7%
Tocantins	4,867	1,529	31.4%	5,129	1,405	27.4%
**Brazil**	**551,678**	**153,284**	**27.8%**	**567,616**	**139,927**	**24.7%**

* 2019 rate applied to 2020 population: the expected value was estimated using the 2019 observed mortality rate in 2019 applied to the 2020 population; Poisson model: the expected value was estimated using Poisson regression. The approach is a Generalized Linear Model regression to estimate deaths in each location, sex, age, race, and week assuming a Poisson distribution. The structure of each model is: $\log(E(Y_i)) = \beta_0 + \beta_1 year$, where $E(Y_i)$ are the expected deaths in a given stratum.

Supplementary Material Table 4 – Sensitivity analysis: Expected deaths, excess deaths, and proportional excess deaths in white, black, and brown populations by methods*. Brazil and states, 2020.

States	White					
	2019 rate applied to 2020 population			Poisson model		
	Expected deaths	Excess deaths	% excess	Expected deaths	Excess deaths	% excess
Acre	720	535	74.3%	997	458	46.0%
Alagoas	4,863	1,375	28.3%	5,018	1,269	25.3%
Amazonas	2,770	1,569	56.7%	2,674	1,627	60.9%
Amapá	712	407	57.2%	892	356	39.9%
Bahia	17,989	3,517	19.5%	18,108	3,275	18.1%
Ceará	13,863	4,843	34.9%	13,815	4,874	35.3%
Distrito Federal	6,725	2,136	31.8%	6,745	2,069	30.7%
Espírito Santo	10,795	2,672	24.8%	11,100	2,461	22.2%
Goiás	15,798	4,418	28.0%	15,806	4,379	27.7%
Maranhão	6,567	2,914	44.4%	6,514	2,975	45.7%
Minas Gerais	67,317	11,269	16.7%	67,145	10,764	16.0%
Mato Grosso do Sul	7,251	1,974	27.2%	7,522	1,800	23.9%
Mato Grosso	6,020	2,319	38.5%	6,230	2,187	35.1%
Pará	6,624	2,956	44.6%	6,933	2,759	39.8%
Paraíba	7,739	2,017	26.1%	7,953	1,860	23.4%
Pernambuco	20,021	4,773	23.8%	19,129	5,359	28.0%
Piauí	4,325	1,045	24.2%	4,510	931	20.6%
Paraná	54,908	7,350	13.4%	54,633	7,026	12.9%
Rio de Janeiro	72,434	17,698	24.4%	72,869	17,128	23.5%
Rio Grande do Norte	8,434	1,744	20.7%	8,495	1,653	19.5%
Rondônia	2,752	973	35.4%	2,841	953	33.6%
Roraima	425	283	66.7%	615	244	39.7%
Rio Grande do Sul	74,490	6,870	9.2%	75,977	5,598	7.4%
Santa Catarina	36,162	5,461	15.1%	36,440	4,920	13.5%
Sergipe	3,471	930	26.8%	3,390	977	28.8%
São Paulo	209,613	24,198	11.5%	207,288	24,209	11.7%
Tocantins	1,832	791	43.2%	1,975	720	36.4%
**Brazil**	**664,619**	**117,037**	**17.6%**	**665,611**	**112,831**	**17.0%**

* 2019 rate applied to 2020 population: the expected value was estimated using the 2019 observed mortality rate in 2019 applied to the 2020 population; Poisson model: the expected value was estimated using Poisson regression. The approach is a Generalized Linear Model regression to estimate deaths in each location, sex, age, race, and week assuming a Poisson distribution. The structure of each model is: $\log(E(Y_i)) = \beta 0 + \beta_1 year$, where $E(Y_i)$ are the expected deaths in a given stratum.

.
